# Effect of Differences in Metabolic Activity of Melanoma Models on Response to Lonidamine plus Doxorubicin

**DOI:** 10.1038/s41598-018-33019-4

**Published:** 2018-10-02

**Authors:** Kavindra Nath, Jeffrey Roman, David S. Nelson, Lili Guo, Seung-Cheol Lee, Stepan Orlovskiy, Kevin Muriuki, Daniel F. Heitjan, Stephen Pickup, Dennis B. Leeper, Ian A. Blair, Mary E. Putt, Jerry D. Glickson

**Affiliations:** 1 Department of Radiology, Perelman School of Medicine, University of Pennsylvania, Philadelphia, PA USA; 2 Center of Excellence in Environmental Toxicology and Department of Systems Pharmacology and Translational Therapeutics, Perelman School of Medicine, University of Pennsylvania, Philadelphia, PA USA; 3 Biostatistics & Epidemiology, Perelman School of Medicine, University of Pennsylvania, Philadelphia, PA USA; 4 Department of Statistical Science, Southern Methodist University, Dallas, TX USA; 5 Department of Clinical Sciences, UT Southwestern Medical Center, Dallas, TX USA; 6 Department of Radiation Oncology, Thomas Jefferson University, Philadelphia, PA USA

## Abstract

Lonidamine (LND), a metabolic modulator, sensitizes DB-1 human melanoma to doxorubicin (DOX) chemotherapy by acidifying and de-energizing the tumor. This report compares the effects of LND on two human melanoma lines, DB-1 and WM983B, which exhibit different metabolic properties. Using liquid chromatography mass spectrometry and Seahorse analysis, we show that DB-1 was more glycolytic than WM983B *in vitro*. ^31^P magnetic resonance spectroscopy (MRS) indicates that LND (100 mg/kg, i.p.) induces similar selective acidification and de-energization of WM983B xenografts in immunosuppressed mice. Over three hours, intracellular pH (pHi) of WM983B decreased from 6.91 ± 0.03 to 6.59 ± 0.10 (p = 0.03), whereas extracellular pH (pHe) of this tumor changed from 7.03 ± 0.05 to 6.89 ± 0.06 (p = 0.19). A decline in bioenergetics (β-NTP/Pi) of 55 ± 5.0% (p = 0.03) accompanied the decline in pHi of WM983B. Using ^1^H MRS with a selective multiquantum pulse sequence and Hadamard localization, we show that LND induced a significant increase in tumor lactate levels (p < 0.01). LND pre-treatment followed by DOX (10 mg/kg, i.v.) produced a growth delay of 13.7 days in WM983B (p < 0.01 versus control), a growth delay significantly smaller than the 25.4 days that occurred with DB-1 (p = 0.03 versus WM983B). Differences in relative levels of glycolysis may produce differential therapeutic responses of DB-1 and WM983B melanomas.

## Introduction

Metastatic melanoma remains a deadly disease with relatively limited and ineffective treatment options. Despite advances in treatment with targeted therapies and checkpoint inhibitors, this disease annually claims approximately 10,000 lives in the US^1^. Targeted therapies exploit common mutations in melanoma such as the BRAF-V600E mutation^1^, but produce only a transient response with median progression-free-survival of 12.3 months^2,3^. On the other hand, checkpoint inhibitors such as anti-PD-1(pembrolizumab) and ipilumimab produce durable responses (over 3 years), but in only ~40% of metastatic melanomas^4–7^. This observation suggests that immunotherapy may have a differential impact depending on the genetic variant of melanoma. There is an additional need for better understanding of how various therapies can be targeted toward different variants of metastatic disease.

In general, cancer cell lines are known to vary substantially in their metabolic characterstics^8^. In particular cancer tends to prefer glycolytic metabolism even in the presence of oxygen (Warburg effect), although this phenotype varies greatly among cancer types. Lonidamine (LND), first introduced in 1979 as an antispermatogenic agent^9^ has limited antineoplastic activity as a single agent but has demonstrated potential in modulating the activities of conventional cancer therapeutic agents. Our recent studies^10^ have shown that LND potently inhibits the mitochondrial pyruvate carrier (MPC) activity in isolated rat liver mitochondria (Ki 2.5 μM) and cooperatively inhibits L-lactate transport by MCT1, MCT2 and MCT4 expressed in *Xenopus laevis* oocytes with K_0.5_ and Hill Coefficient values of 36–40 μM and 1.65–1.85, respectively. LND inhibits the succinate-ubiquinone reductase activity of respiratory complex II without fully blocking succinate dehydrogenase activity. LND also induces cellular reactive oxygen species through complex II, and has been reported to promote cell death by suppression of the pentose phosphate pathway, which resulted in inhibition of NADPH and glutathione generation. We concluded that MPC inhibition is the most sensitive anti-tumor target for LND, with additional inhibitory effects on MCT-mediated L-lactic acid efflux, complex II and glutamine/glutamate oxidation^11^. Metabolically, LND inhibits respiration^11^, export of lactic acid^11,12^, and production of ATP^11–14^. Together these metabolic effects acidify^11–14^ and de-energize^11–14^ the affected cells. Perhaps because cancer cells are more dependent on glycolysis and normal tissue is more dependent on respiration, LND selectively perturbs tumor cells with minimal effects on tissues such as liver, muscle or brain^12,15^. LND has chemotherapeutic^12–17^ activity as well as the ability to sensitize tumors to hyperthermia^18–22^ and radiation^23,24^. Clinical trials have evaluated LND’s toxicity and efficacy against several malignancies^25–27^ and benign prostate hyperplasia^28^.

Recently, we showed that LND selectively acidifies, de-energizes, and sensitizes DB-1 melanoma to doxorubicin (DOX) chemotherapy^14^. During a three hour experiment on DB-1 xenografts in nude mice treated with LND (100 mg/kg, i.p.), the intracellular pH (pHi) fell from 6.90 to 6.33 while the extracellular pH (pHe) was more stable dropping only from 7.0 to 6.80^12^. Simultaneously, the β-NTP/Pi ratio (beta-nucleoside triphosphate/ inorganic phosphate molar ratio (which measures the bioenergetic state of the tumor), as measured by ^31^P magnetic resonance spectroscopy (MRS) decreased monotonically by 67% over a three hour period. In addition, LND pretreatment potentiated the antitumor activity of DOX (10 mg/kg, i.v.). Compared to control, DOX alone yielded a growth delay of 5.4 days while DOX combined with LND yielded a growth delay of 28.6 days^12^. This combination of LND and DOX produced 98% cell kill with one dose^14^.

Our earlier study raised questions about whether the dramatic enhancement of the response of DB-1 to DOX^14^ was consistent across different melanoma cell lines, or whether there were specific metabolic characteristics of the DB-1 line that made it particularly susceptible to treatment with LND. In the current study, we contrast the response of DB-1 to the WM983B human melanoma model. WM983B is an established human melanoma cell line and xenograft model that, like DB-1 expresses BRAF-V600E, a characteristic mutation in melanoma which is selectively targeted by the BRAF kinase inhibitor vemurafenib^16^. We evaluated the metabolic and therapeutic effects of LND on WM983B both *in vitro* and *in vivo*. We also considered any differential responses to LND between WM983B and DB-1 cells and xenografts and used our understanding of the mechanism of action of LND action to interpret these differential responses. This study adds to our understanding of improvements to cancer therapy in two ways. First we are able to assess the metabolic response to a chemotherapy modulator *in vivo* using a unique non-invasive MR technique. Secondly, our work provides insight on the mechanistic basis for differences in the effect of this metabolic modulator on response to chemotherapy of the two melanoma lines.

## Results

### Liquid Chromatography Mass Spectrometry (LC-MS) Metabolite Quantification in Cells

**Figure 1:**
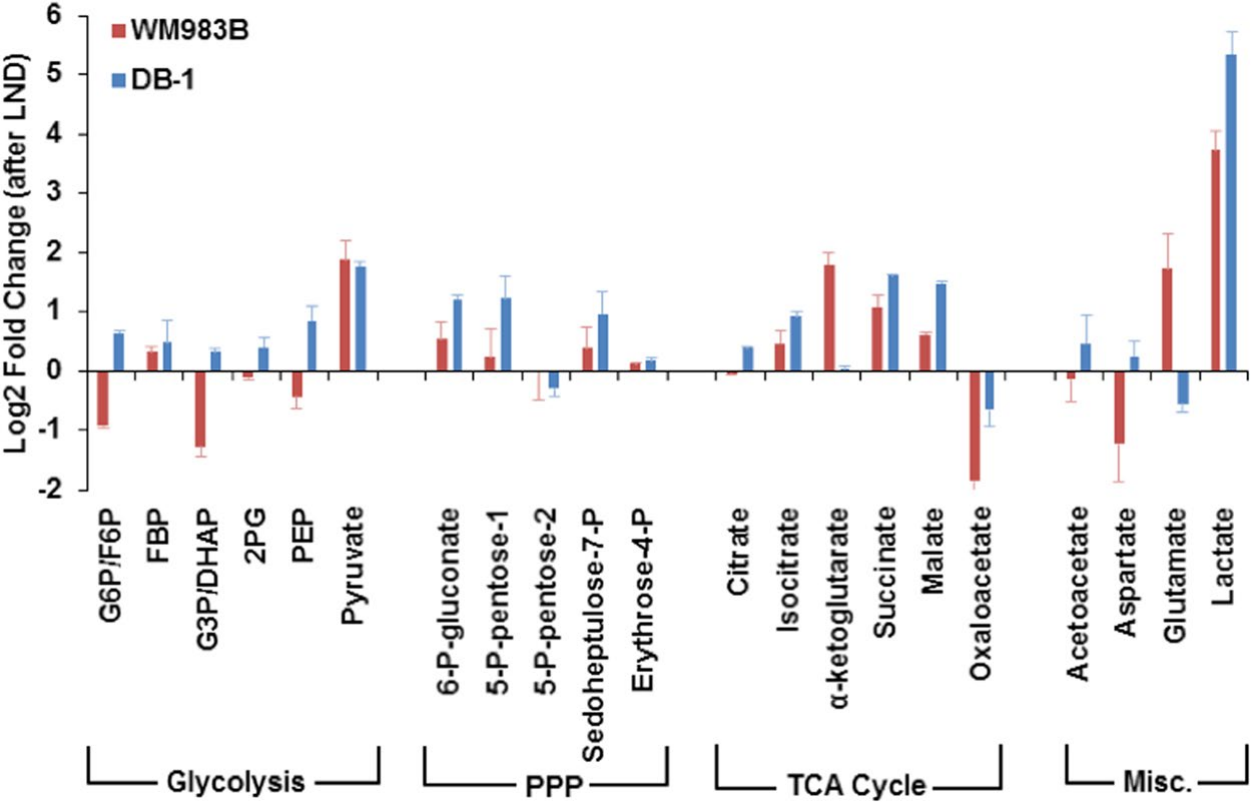
Lonidamine (LND) induces different metabolic responses in WM983B and DB-1 human melanoma cells one hour post treatment. For a given metabolite and cell line, we calculate fold change as the intracellular metabolite amount in the LND group divided by the mean metabolite amount in the CTRL (DMSO sham) group. These fold changes are represented as log base 2. Data shown as mean ± SD.

LND induces different metabolic responses in DB-1 and WM983B melanoma cell lines. In particular, increases in lactate were noticeably higher in DB-1 while increases in glutamate and  α-ketoglutarate were noticeably higher in WM983B (Fig. 1). Aspartate, oxaloacetate, G3P/DHAP and G6P/F6P declined sharply in WM983B in contrast to DB-1 where these metabolites exhibited only small changes.

### Seahorse and YSI Cell Metabolism Assays

**Figure 2:**
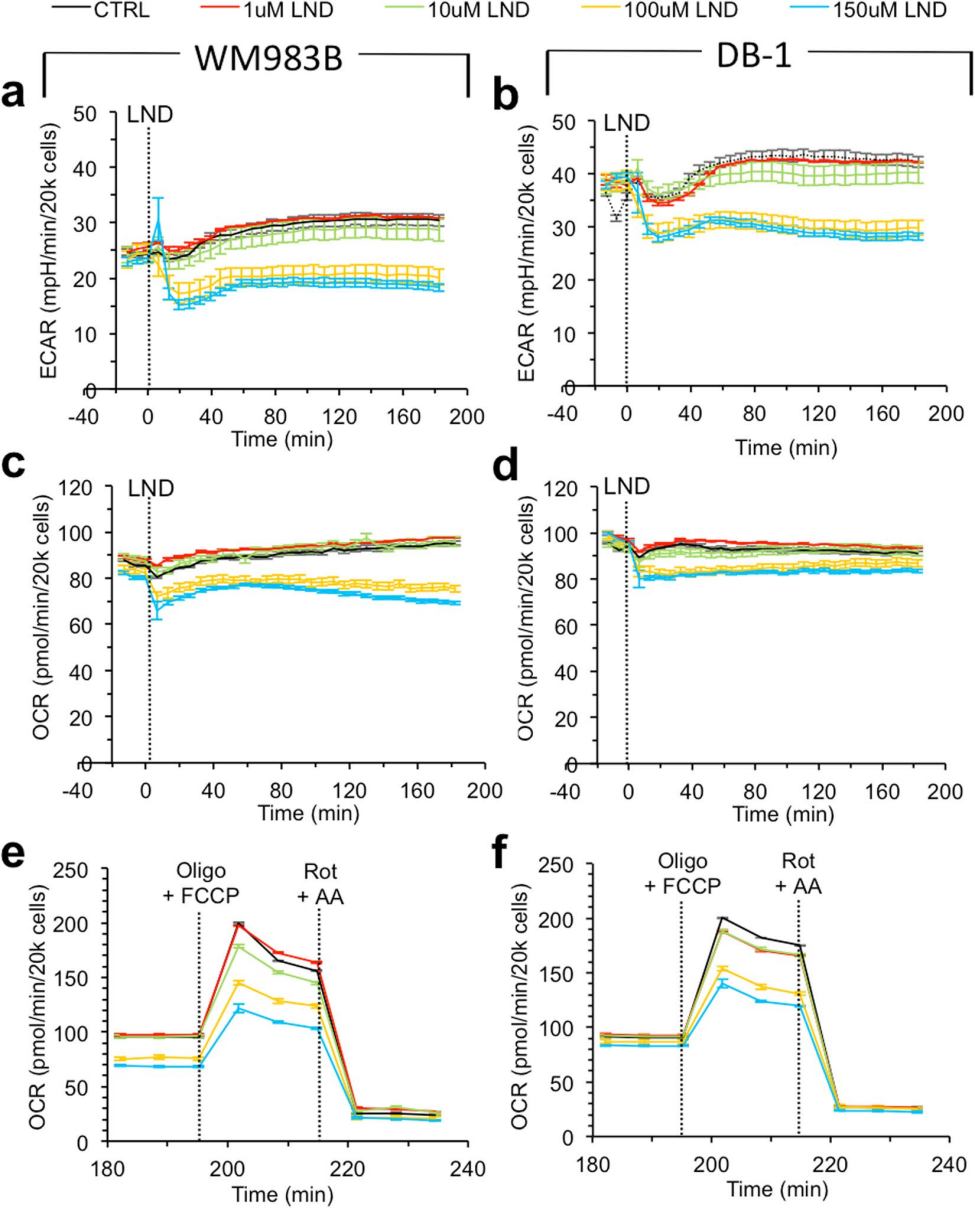
Lonidamine (LND) induces a concentration-dependent decrease in metabolic rates in WM983B and DB-1 human melanoma cells. For WM983B and DB-1, respectively, (**a**,**b**) show extracellular acidification rate (ECAR) vs. time after LND treatment. (**c**,**d**) show oxygen consumption rate (OCR) vs. time after LND treatment. (**e**,**f**) show OCR vs. time after LND treatment upon addition of oligomycin (Oligo) with carbonyl cyanide 4-(trifluoromethoxy) phenylhydrazone (FCCP), then rotenone (Rot) with antimycin A (AA). Data shown as mean ± s.e.m.

DB-1 is a more glycolytic melanoma cell line than WM983B. DB-1 has significantly higher basal extracellular acidification rate (ECAR) (p < 0.001) and lower oxygen consumption rate to extracellular acidification rate ratio (OCR)/ECAR ratio (p < 0.001) (Table 1, Fig. 2).

**Table 1:** WM983B cells are less glycolytic than DB-1 cells. We display metabolic parameters derived from *in vitro* Seahorse and YSI metabolic assays, including oxygen consumption rate (OCR) and extracellular acidification rate (ECAR). Data shown as mean ± S.D.

Metabolic Parameter	WM983B		DB-1		*p*-value
	mean ± S.D.	n	mean ± S.D.	n	
Basal OCR^1^	90 ± 10	23	94 ± 5	23	<0.001
Stressed OCR^1^	200 ± 20	3	200 ± 10	3	0.989
Basal ECAR^2^	24 ± 2	23	38 ± 2	23	<0.001
Stressed ECAR^2^	61 ± 3	3	74 ± 5	3	0.056
Basal OCR/ECAR Ratio^3^	3.5 ± 0.3	23	2.5 ± 0.2	23	<0.001
Glucose Consumption^4^	4 ± 2	16	7 ± 3	11	0.003
Lactate Production^4^	7 ± 3	16	14 ± 5	11	0.003

^1^pmol/2 × 10^4^ cells/min; ^2^mpH/2 × 10^4^ cells/min; ^3^pmol O_2_/mpH; ^4^nmol/10^6^ cells/min.

**Figure 3:**
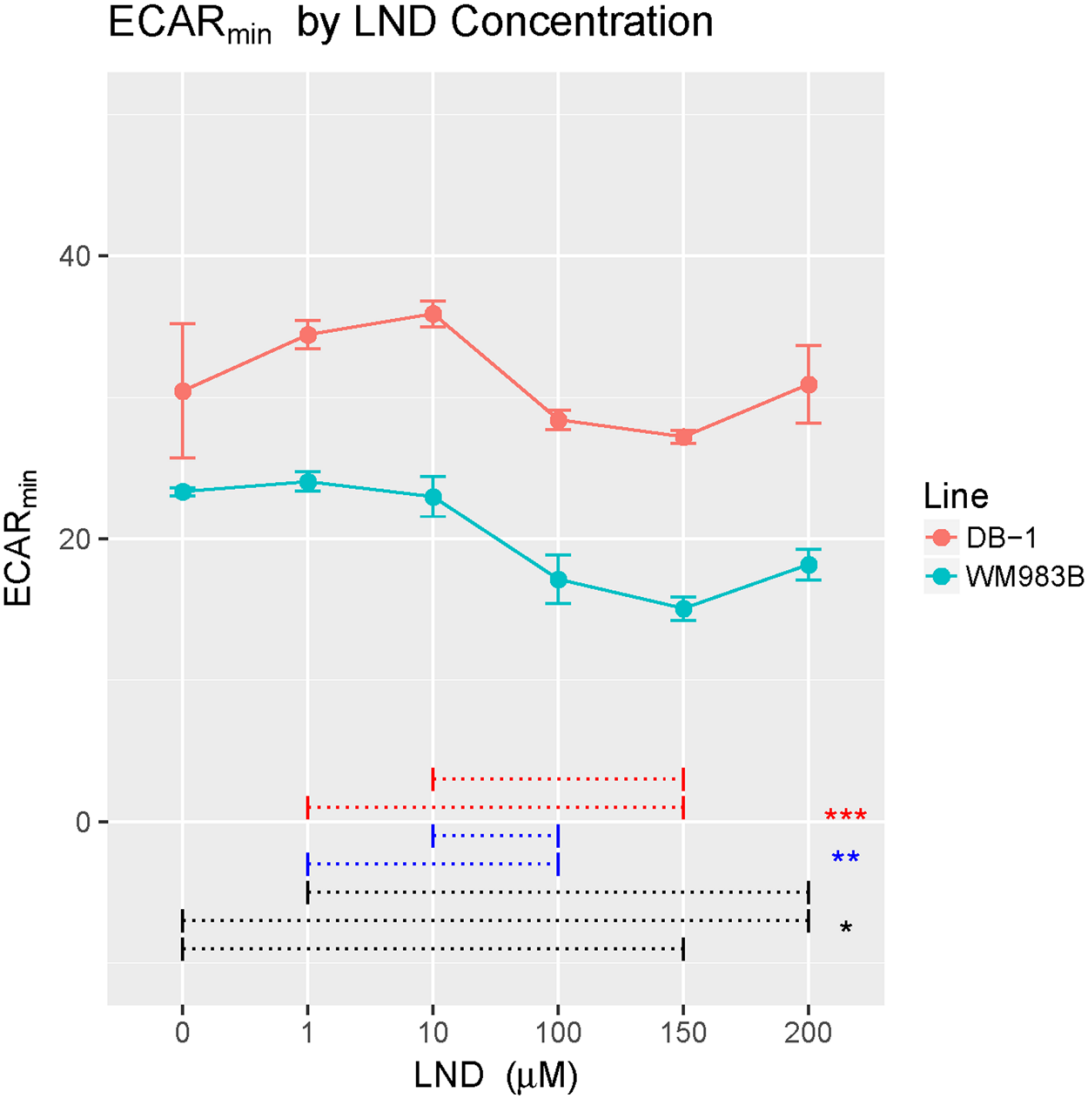
Lonidamine (LND) induces a concentration-dependent acute inhibition of extracellular acidification in DB-1 and WM983B human melanoma cells. We show ECAR_min_ the lowest measured extracellular acidification rate within the first 60 minutes of LND treatment, against LND concentration (in µM). ECAR_min_ for DB-1 was significantly larger than for WM983B (p < 0.001 F_1,34_ = 145) and there were significant effects as well for LND concentration (p < 0.001, F_5,34_ = 9.2). Dotted lines connect LND concentrations of either 0, 1 or 10 uM with higher concentrations where there were significantly different mean levels of ECAR_min_. (adjusted p < 0.001 ***p < 0.001**p < 0.05*) e.g. mean ECAR_min_ differed significantly between 0 & both 150 and 200 uM LND (p < 0.05).

LND induces decreases in metabolic rates in both DB-1 and WM983B. LND induces ECAR inhibition in both DB-1 and WM983B within the first hour of treatment (Fig. 2a,b). Figure 3 shows the minimum value of ECAR as a function of LND dose. The acute response of ECAR to LND is concentration-dependent with considerable inhibition occurring at doses of 100 µM and greater (Fig. 3). DB-1 consistently exhibited larger mean values of ECAR_min_ (p < 0.001). There was no evidence of modification of these differences by LND concentration (p = 0.58). LND induces inhibition of both OCR and ECAR over a threehour period post-treatment, with greater doses inducing greater reductions (Fig. 2a–d). The LND inhibition of metabolic rates persists after stressing the cells with oligomycin and carbonyl cyanide 4-(trifluoromethoxy) phenylhydrazone (FCCP) then rotenone (Rot) with antimycin A (AA) (Fig. 2e–f).

### Magnetic Resonance Spectroscopy of Melanoma Xenografts in Mice

**Figure 4:**
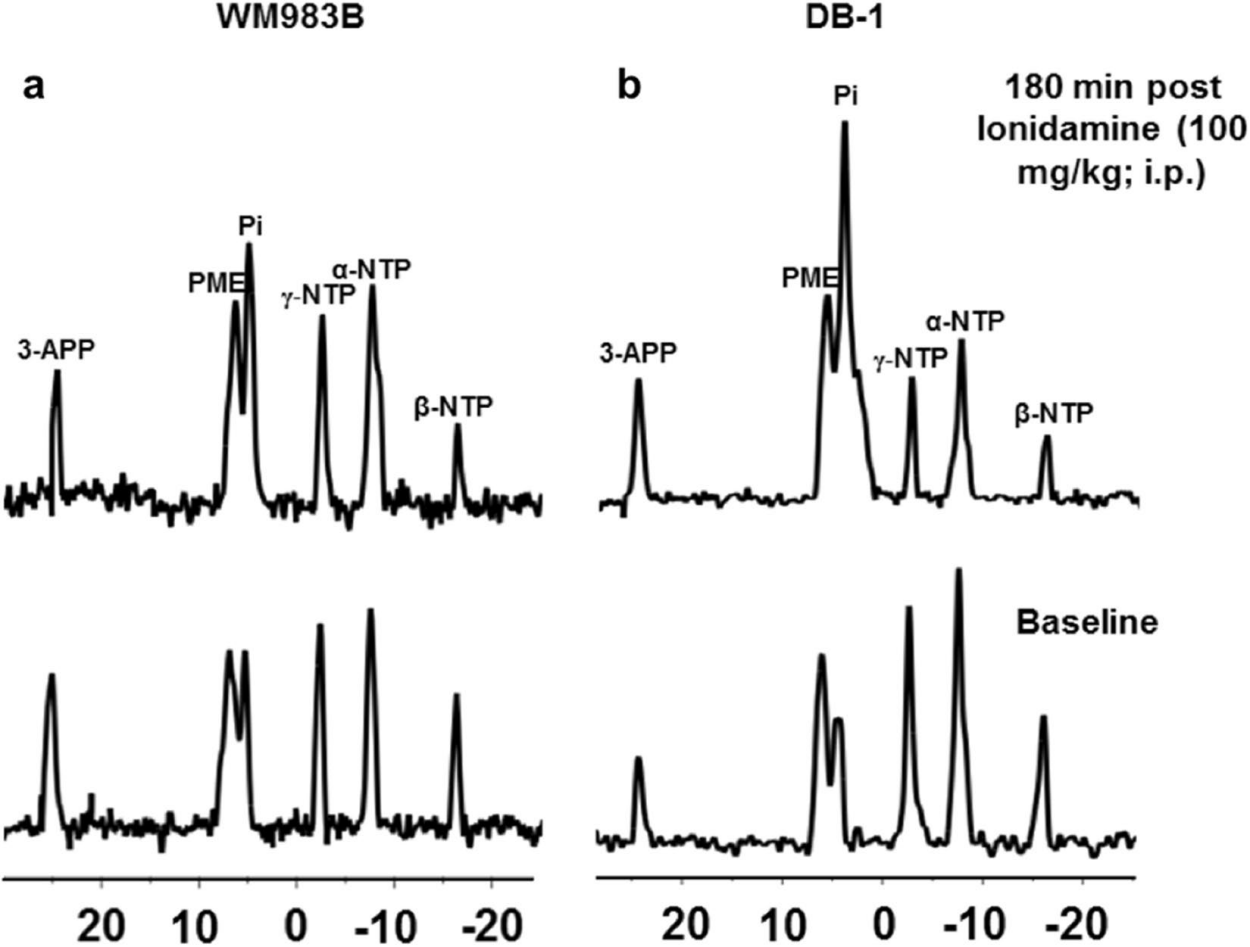
Lonidamine (LND) causes a decrease in bioenergetics in WM983B xenografts. We show representative *in vivo* localized (Image Selected *In vivo* Spectroscopy; ISIS) Phosphorus-31 magnetic resonance spectroscopy (^31^P MRS) spectra of (**a**) WM983B and (**b**) DB-1 xenografts^43^. In each set, the lower spectrum represents the baseline and upper represents 180 minutes post-LND treatment. We mark resonances for 3-aminopropylphosphonate (3-APP), phosphomonoesters (PME), inorganic phosphate (Pi), γ-nucleoside-triphosphate (γ-NTP), α-nucleoside-triphosphate (α-NTP), and β-nucleoside-triphosphate (β-NTP). A decrease in β-NTP levels with corresponding increase in Pi indicates impaired energy metabolism.

For WM983B xenografts, exposure to LND decreased the bioenergetic status and selectively acidified the tumor. We show representative localized ^31^P MR spectra before and after LND treatment in WM983B (Fig. 4a) and DB-1 melanoma xenografts (Fig. 4b). Of note, LND caused a decrease in β-NTP with a simultaneous increase in Pi. The β-NTP/Pi remained low at 55 ± 0.05% of the baseline level for up to 120 min (differences from baseline were statistically significant (p < 0.05) at t = 100, 120, 160) in WM983B melanoma xenografts. The integrated intensity of β-NTP and Pi maximum decreased by 28% and increased by 68%, respectively, after LND administration.

**Figure 5:**
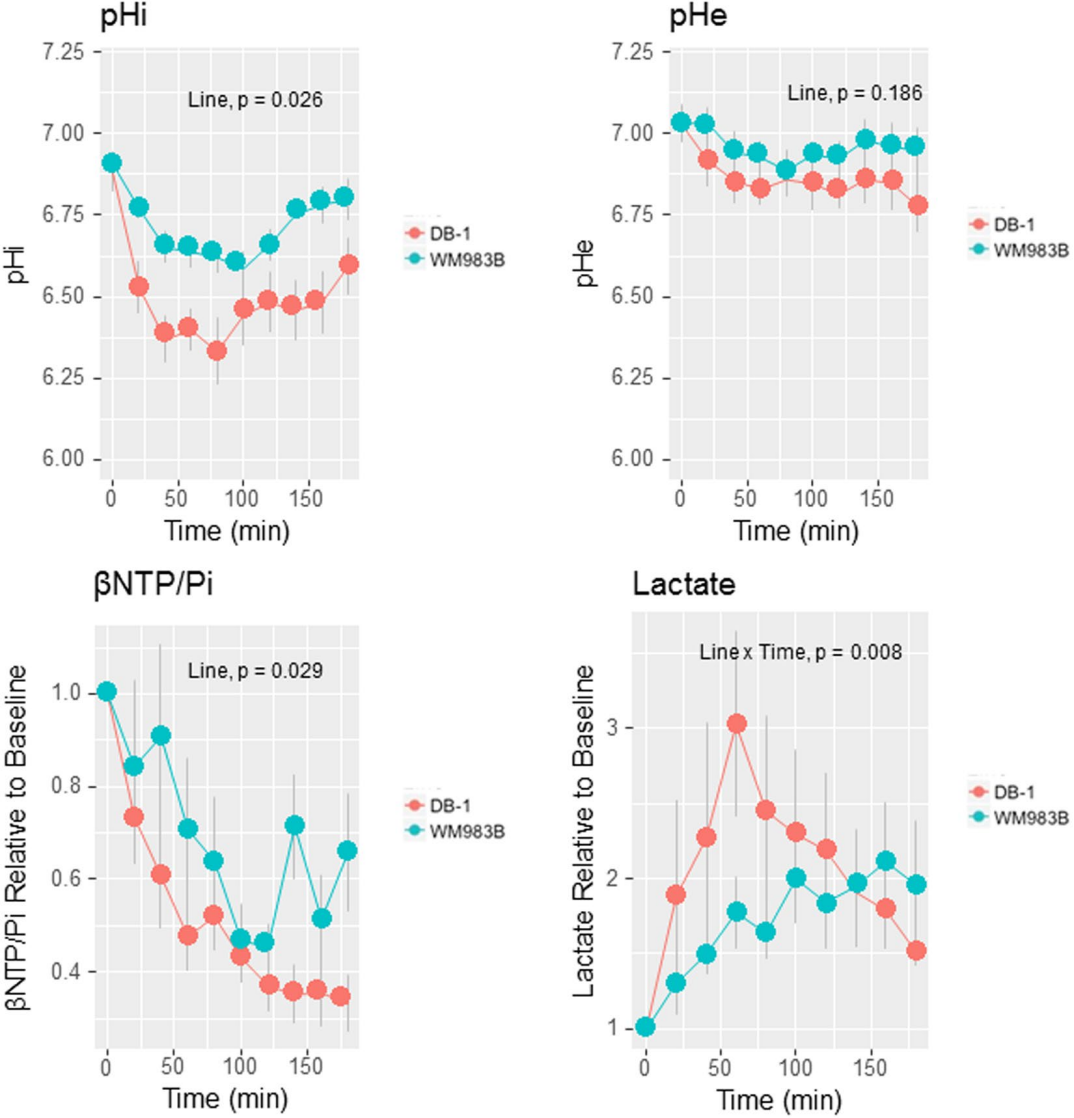
Lonidamine (LND) causes simultaneous acidification and de-energization of WM983B human melanoma xenografts. For comparison, we include previously published results for DB-1 xenografts^12^ Intracellular pH (pHi) differed by melanoma line (p = 0.036, F_1,20_) while no differences between cell lines were detected for pHe. Bioenergetics (ratio of β-nucleoside triphosphate to inorganic phosphate (β-NTP/Pi) differed by tumor line (p = 0.029, F_1,20_) while lactate showed differential effects by  tumor as a function of time (p = 0.008, F_1,48_) (d) shows tumor lactate relative to baseline (n = 5). Data shown as mean ± s.e.m.

After LND treatment, the tumor pHi of WM983B xenografts decreased from 6.91 ± 0.03 to 6.59 ± 0.05 (p < 0.01 for all times except t = 180 min), reaching its lowest point at 100 minutes post- treatment (Fig. 5). In contrast, the tumor pHe of this tumor declined only slightly, from 7.03 ± 0.05 to 6.89 ± 0.06 (difference in pHe not statistically significant at any time point) (Fig. 5). Simultaneously, lactate concentrations were significantly elevated (p < 0.01) at 40 min after LND administration (Fig. 5). Since the pHi decreased more than pHe, and since extracellular volume fraction is ~40% in experimental tumors^29,30^, we conclude that most of the lactate increase was primarily intracellular. 3-aminopropylphosphonate (3-APP) (control sham) did not change tumor pHi, pHe, or lactate levels over a three hour period (data not shown).

### Tumor Growth Delay Studies with Doxorubicin

**Figure 6:**
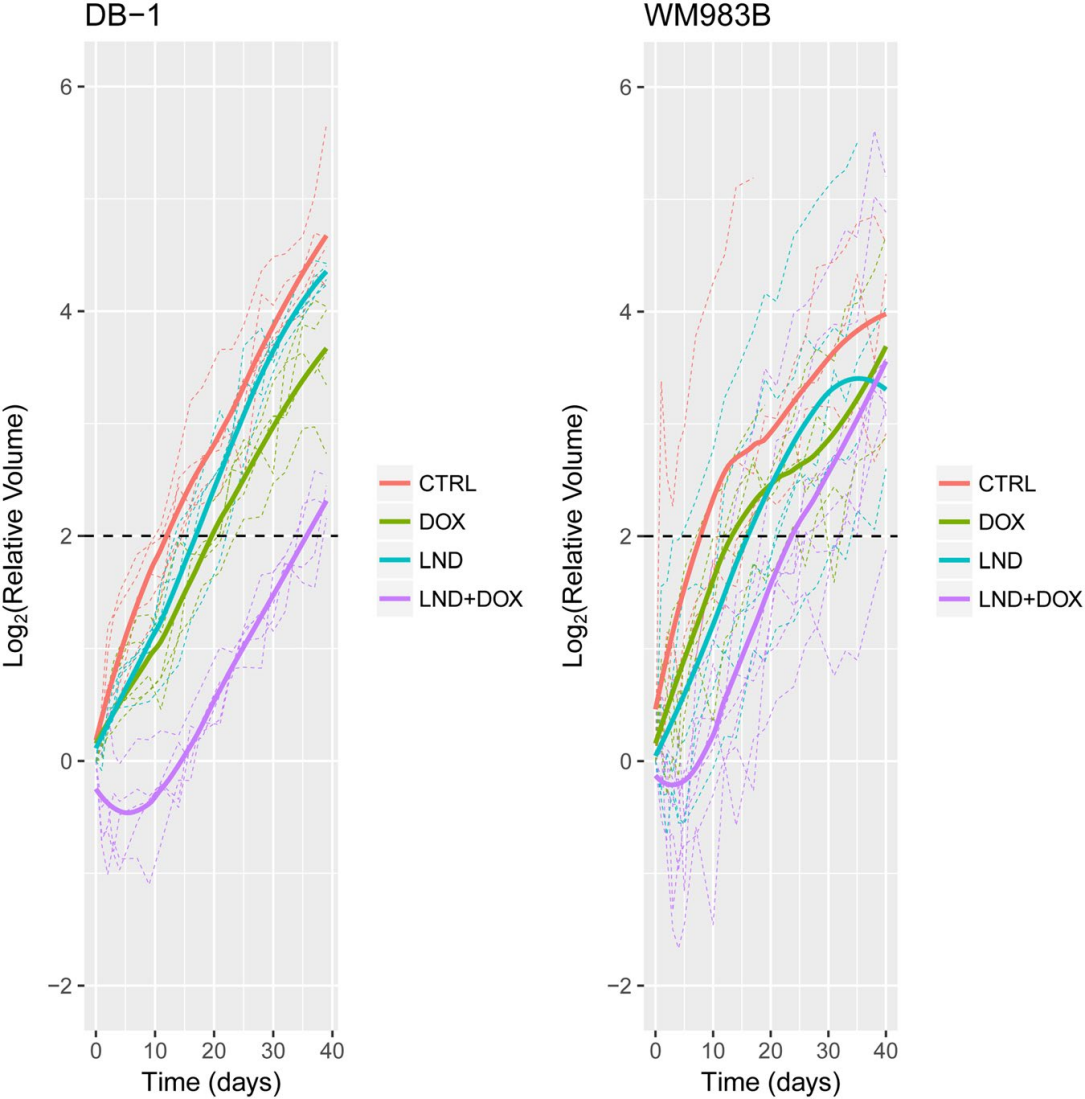
Lonidamine (LND) sensitizes both DB-1 and WM983B human melanoma xenografts to doxorubicin (DOX). On Day 0, nude mice with WM983B xenografts received treatment as follows: Control (sham i.p. tris/glycine + sham i.v. PBS), LND (100 mg/kg i.p.), DOX (10 mg/kg i.v.), LND + DOX). The plots show the growth measurements for each animal relative to tumor volume at Day 0. Dark lines are the Loess-smoothed curves for each treatment group. The dashed line at 2 indicates 4 doublings. The data in the controls up to 12 days were used to determine doubling time. The data for DB-1 were re-analyzed from a previous publication in order to be comparable to the analysis for WM983B^14^.

In the control groups, tumors for the WM983B and DB-1 melanoma xenografts doubled at very similar rates (5.52 days for DB-1 and 5.37 days for WM983B). As reported earlier for DB-1, LND provides a dramatic potentiation of DOX toxicity. Notably, in DB-1 DOX administered alone induced a growth delay of 8.1 (90% CI 21.0, 29.1) days compared to control, whereas LND + DOX induced a growth delay of 25.4 (90% CI 6.1, 10.2) days versus control (Fig. 6, Table 2). Compared to control, percent survival of WM983B was 36.2% (90% CI 27.2, 47.2) for DOX alone and 4.1% (90% CI 2.5, 7.4) for DOX + LND. WM983B was also potentiated by LND but not to the same extent as DB-1. For WM983B, no significant growth delay was noted for DOX alone (p = 0.20, 90% CI −2.3, 11.1) whereas with LND + DOX, a growth delay of 13.7 days was observed (90% CI 7.6, 20.3, p = 0.005) (Fig. 6, Table 2). Compared to control, percent survival in WM983B was also not significantly reduced (p = 0.15, 90% CI, 15.4, 126.4) with DOX alone but fell to 17.1% (90% CI 3.5, 45.9 p < 0.001) with LND + DOX (Fig. 6, Table 2). For tumors treated with a combination of LND + DOX, the mean growth delay for DB-1 of 25.4 days was significantly longer than for WM983B (p = 0.032). No other differences between the cell lines achieved statistical significance (Fig. 6, Table 2).

**Table 2:** Lonidamine (LND) induces a lesser potentiation to doxorubicin (DOX) in WM983B human melanoma xenografts compared to DB-1 human melanoma xenografts. We show estimated growth delay (T-C), doubling time (T_d_), and surviving fraction as percent by treatment arm in WM983B and DB-1 xenografts following LND administration. The data for DB-1 were re-analyzed from a previous publication in order to be comparable to the analysis for WM983B^14^.

Treatment Group (versus Control)	Growth Delay (days)* (Mean with 90% CI)			Surviving Fraction (%)* (Estimate with 90% CI)			Number of Animals (n)	
	DB-1	WM983B	p**	DB-1	WM983B	p**	DB-1	WM983B
Lonidamine (LND)	4.4 (1.5, 7.4)	7.9 (0.1, 16.8)	—	57.8 (38.8, 83.2)	36.0 (6.1, 99.2)	—	4	5
*p*-value*	0.047	0.108	0.55	0.026	0.053	0.82		
Doxorubicin (DOX)	8.1 (6.1, 10.2)	3.7 (−2.3, 11.1)	—	36.2 (27.2, 47.2)	62.1 (15.4, 126.4)	—	5	5
*p*-value*	<0.001	0.201	0.34	<0.001	0.153	0.79		
LND + DOX	25.4 (21.0, 29.1)	13.7 (7.6, 20.3)	—	4.1 (2.5, 7.4)	17.1 (3.5, 45.9)	—	5	8
*p*-value*	< 0.001	0.005	0.032	< 0.001	< 0.001	0.90		

In the control, doubling times were 5.52 days for DB-1 and 5.38 days for WM983B.

^*^p-value for one-sided test comparing each treatment group to control ^**^Two-sided *p*-value comparing the two cell lines.

LND produced minor systemic toxicity to our mice with WM983B tumors. Mice that received LND alone lost 2.4% of their body weight by day 1 and returned to baseline by the next day. Mice that received DOX alone lost 13% of their body weight within 11 days of injection and then showed no further weight loss for at least 40 days. Mice that received both LND and DOX experienced similar weight loss to mice that received DOX alone (data not shown).

## Discussion

DB-1 and WM983B human melanoma xenografts appear to respond similarly to LND, although the response was somewhat muted in WM983B. LND selectively acidified WM983B tumors causing a noticeable decrease in pHi and a minimal drop in pHe. Simultaneously, LND de-energized WM983B tumors, causing a decrease in β-NTP and an increase in inorganic phosphate. In addition, LND pre-treatment potentiated single-dose DOX to produce cell kill that was significantly greater than that of DOX alone. Compared to DB-1 melanoma xenografts, WM983B displayed less acidification, de-energization, and potentiation in chemotherapy response. These two cell models were chosen based on the diverse nature of their metabolomics, but with similar oncogenic phenotypes both carrying the V600E BRAF mutation.

LND-induced acidification likely contributes to DOX sensitization by inhibiting the MCT and MPC resulting in tumor acidification. LND may cause free alkaline DOX (pKa~8.5) to be more readily retained in the acidified tumor cells (cation trapping)^14^. Since WM983B melanoma xenografts are less glycolytic and less acidified by LND than DB-1 melanoma xenografts, they may experience less cation trapping and thus less response to DOX^31^.

Interestingly, WM983B melanoma xenografts seem to upregulate glutaminolysis after LND treatment, to compensate for the energy loss associated with blocking MPC. We determined that glutaminolysis was being employed for anaplerotic flux into the TCA cycle based on the LC-MS data (Fig. 1) showing increases in intermediary metabolites immediately downstream of glutamine, specifically glutamate and α-ketoglutarate. This effect was specific to WM983B, which is the less bioenergetically affected of the two melanoma cell lines, so we believe that this anaplerotic flux plays a role in its diminished response. Glutamine is required for proliferating tumor cells as it participates in bioenergetics, supports cell defenses against oxidative stress and complements glucose metabolism in the production of macromolecules^32^. This metabolic pathway may be the escape mechanism used by WM983B melanoma xenografts and should be studied further.

By trapping lactate inside tumor cells, we wanted to exploit this natural preference of tumors for glycolytic metabolism. Glycolysis selectively induces intracellular acidification of the tumor through accumulation of lactic acid even under aerobic conditions. This selective tumor acidification and de-energization (caused by inhibition of pyruvate uptake by mitochondria and by inhibition of complex II) can greatly enhance tumor response to chemotherapeutic agents such as N-mustards and DOX^12–17^. Others have shown that LND sensitizes tumors to hyperthermia^18,20–22^, radiotherapy^23,24^ and photodynamic therapy^33^ by a variety of different mechanisms. Through cation trapping, LND may enhance the uptake and retention of various other cancer drugs including anthracyclines, which are weakly basic. Swietach *et al*. have shown the importance of pHi in determining the uptake and efficacy of DOX on the basis of pH-partitioning across cell-surface and pH-sensitivity of binding to nuclear sites which maintains DOX in a slowly-releasable form^34^. Our study demonstrates that reversal of the pH gradient caused by LND should increase uptake of weak bases such as DOX by melanoma cells. There is no reason to believe that the acidification and reversal of the pH gradient seen with these melanoma cell lines would not occur with other tumor types. By taking advantage of the altered physiological phenotype of the tumor, the addition of LND to traditional chemotherapy modalities could result in improved therapeutic gain.

LND-induced acidification also inhibits key DNA-repair enzymes such as O^6^-alkylguanine DNA alkyltransferase, so LND can also potentiate therapies such as temozolomide treatment that damage the DNA of cancer cells^17^. In addition, by inhibiting tumor respiration, LND may increase tumor oxygenation and thus increase the effect of reactive oxygen species that play an important role in the activity of some anti-cancer agents most notably radiation therapy that have a free-radical intermediate in their action. We found that while LND alone and DOX alone had minimal effects on melanoma growth the combination of these agents administered according to a schedule based on MRS measurement of tumor pHi, substantially decreased the growth rate of this highly malignant tumor. This could provide a method for improved systemic therapy of this deadly cancer.

LND-induced de-energization may also contribute to DOX sensitization. Multi-drug resistance appears to be mediated by an energy dependent mechanism whereby a p-glycoprotein pumps cytotoxic drugs out of the tumor^14,34^. By blocking the MPC and de-energizing tumor cells, LND may reduce tumor ATP levels and thereby reduce multi-drug resistance. Because WM983B cells are inherently less energetic than DB-1 cells *in vitro*, we predict that LND should induce less de-energization in WM983B xenografts and thus be less effective in potentiating the toxic effects of DOX. This is indeed what we observed. At least conceptually, this model has potential for translation to clinical settings. Notably, we now have instruments and methodologies to non-invasively monitor tumor metabolism and energetics. Bioenergetic measurements could form the basis of predictions about the response of the tumor to an energetically demanding treatment. Melanoma therapy has been recently directed toward immunotherapy and specific BRAF V600E inhibitors. Although these novel targeted therapies are promising, curative systemic treatment of melanoma remains elusive for the majority of melanoma patients. It is reasonable to think that information about metabolism could also be highly useful in monitoring and predicting response to other treatment modalities such as BRAF inhibitors and immunotherapy.

## Materials and Methods

### Reagents

DMEM, glutamine, HEPES, penicillin-streptomycin, trypsin-EDTA, DPBS, sodium pyruvate, and HBSS were purchased from Thermo Fisher Scientific (Waltham, MA, USA; Invitrogen brand). FBS was purchased from HyClone Laboratories, Inc. (Logan, UT, USA). Glucose, DMSO, methanol, water, phenylhydrazine hydrochloride, trizma base, and glycine were purchased from Sigma-Aldrich (St. Louis, MO, USA). T-75 flasks, trypan blue, and 10-cm culture dishes were purchased from Corning Life Sciences (Tewksbury, MA, USA; Falcon brand). LND and 3-APP were purchased from Santa Cruz Biotechnology, Inc. (Santa Cruz, CA, USA). Seahorse XF Base Medium, oligomycin, FCCP, rotenone, and antimycin A were purchased from Seahorse Bioscience, Inc. (North Billerica, MA, USA). DOX was purchased from Midwest Veterinary Supply (Burnsville, MN, USA).

Male athymic nude mice (01B74) 4–6 weeks of age were purchased from the National Cancer Institute (Frederick, MD, USA) and housed in microisolator cages with access to water and autoclaved mouse chow *ad libitum*. For *in vivo* experiments, the following accessories were purchased from the following vendors: a respiration pillow from SA Instruments Inc. (Stony Brook, NY, USA; Model 1025), water pad heater from Gaymar Industries, Inc. (Orchard Park, NJ, USA), catheters from Tyco Healthcare (Milwaukee, WI, USA), restrainer from Braintree Scientific, Inc. (Braintree, MA, USA), calipers from Bel-Art Products (Wayne, NJ, USA), and scale from H&C Weighing Systems (Columbia, MD, USA; Acculab PP401).

### Cell Culture

Our culture medium for WM983B cells consists of DMEM supplemented with 10% FBS, 25 mM glucose, 4 mM glutamine, 10 mM HEPES, 100 units/mL penicillin, and 100 µg/mL streptomycin. We grew WM983B cells in T-75 flasks with this culture medium and incubated the cells at 37 °C and 5% CO_2_. We passaged the cells at 80% confluence using 0.05% trypsin-EDTA for 2 minutes, and split the cells 1/20 into new flasks. For experiments requiring specific cell numbers, we counted the cell suspensions with a hemocytometer using trypan blue stain. We cultured DB-1 cells as described previously^12,14–17^.

### LC-MS Metabolite Quantification

DB-1 or WM983B cells were grown in 10-cm culture dishes until reaching 80% confluency. Medium was replaced with fresh culture medium containing LND (150 µM) or DMSO vehicle (0.15%) and cells incubated for one hour. Next, cells were washed three times with cold DPBS and scraped with 750 µL methanol: water (80:20) containing 1 mg phenylhydrazine hydrochloride and pulse-sonicated for 20 seconds. Extracts were centrifuged at 17,000 × g and supernatants retained. Supernatants were dried with nitrogen gas and resuspended in mobile phase for LC-MS analysis.

### Seahorse and YSI Metabolic Assays

I*n vitro* oxygen consumption and extracellular acidification rates were measured with the Seahorse XFe96 Analyzer. For our custom protocol, 2 × 10^4^ cells were seeded per well and assay medium was made by supplementing Seahorse XF Base Medium with 5 mM glucose, 2 mM L-glutamine, and 1 mM sodium pyruvate. First three basal OCR and ECAR measurements (3-0-3 mix-wait-measure cycle) were performed. Next, LND (0, 1, 10, 100, or 150 µM; 0.2% DMSO) was injected and measurements were made for three hours. Then, 1 µM FCCP and 1 µM oligomycin were added, and three measurements were made. Lastly, 0.5 µM rotenone and 0.5 µM antimycin A were added followed by three measurements. After this experiment, cells were typsinized with 0.05% trypsin-EDTA and counted with a hemocytometer. Simultaneously, glucose and lactate concentrations in the medium were measured using a YSI 2300 STAT Plus Glucose & Lactate Analyzer calibrated with YSI 2747 dual standard. To calculate the glucose consumption and lactate production rates, we assumed that the cells were in the logarithmic growth phase and that the change in the amounts of metabolites was proportional to the number of cells in the flask. Under these assumptions, glucose consumption and lactate production rates were calculated with the following equation,

1 $$\dot{{\rm{A}}}=\frac{({\rm{A}}({\rm{t}})-{\rm{A}}(0))\mathrm{ln}(\frac{{\rm{N}}({\rm{t}})}{{\rm{N}}(0)})}{{\rm{t}}({\rm{N}}({\rm{t}})-{\rm{N}}(0))}$$

where N(t) is the number of cells at time t after seeding, N(0) is the number of cells seeded, A(t) is the amount of a given metabolite in the well at time t, and $\dot{{\rm{A}}}$ is the characteristic metabolic rate. Note that if $\dot{{\rm{A}}}$ is positive, the metabolite is net-produced; if $\dot{{\rm{A}}}$ is negative, A is net-consumed.

### Human Melanoma Xenografts in Nude Mice and *in vivo* Doubling Time Calculation

WM983B xenografts were grown in nude mice as described in our previous publications with DB-1 melanoma xenografts^12,14–17,35^. Briefly, 1 × 10^6^ cells in 100 µL HBSS were inoculated subcutaneously in the right flank of nude mouse. *In vivo* xenograft doubling times were calculated by measuring tumor growth in untreated mice with calipers, using the formula for the volume of a half-ellipsoid, *V* = (π/6) (*l* × *w* × *d*), where *l* is the tumor’s length, *w* is the tumor’s width, and *d* is the tumor’s depth. Tumor doubling times were calculated from the slopes of the log-linear portion of the tumor growth curves (determined by linear regression analysis) using the formula *t*_*d*_ = 0.3010 /*m*, where *m* is the slope.

### Magnetic Resonance (MR) Experiment for pH Measurement and Bioenergetics Status Estimation

MR experiments were performed on a 9.4 T/31-cm horizontal bore Varian system. Changes in pHi and pHe were measured using ^31^P MRS by positioning the tumor in a dual-frequency (^1^H/^31^P) slotted-tube resonator (10 mm diameter) built in-house. For ^31^P MRS, the Image Selected *In* vivo Spectroscopy (ISIS) technique was used with the following parameters: Hyperbolic Secant-Adiabatic Fast Passage (HS-AFP) slice-selective inversion pulses with 2.5 ms length, 296 scans with a radiofrequency pulse width of 60 µs, corresponding approximately to a 90° flip angle; sweep width, 12 kHz; 512 data points; TR = 4 s.

Lactate was measured using ^1^H MRS by positioning the tumor in a single frequency (^1^H) slotted-tube resonator (13 mm inner diameter, 15 mm outer diameter, 16.5 mm depth) built in-house. For ^1^H MRS, a slice-selective, double-frequency, Hadamard-selective, multiple quantum coherence transfer pulse sequence was used to detect lactate and to filter out overlapping lipid signals^36^. Following acquisition parameters were used: sweep width = 4 kHz; 2048 data points; TR = 8 s; 128 scans.

Magnet was shimmed globally until the water line-width of the tumor monitored via the ^1^H channel reached 60–70 Hz. The point resolved spectroscopy (PRESS) sequence was used to shim the ISIS tumor voxel of 250–300 mm^3^ volume covering the entire tumor depending on tumor size to 30–40 Hz line width.

To prepare tumor-bearing mice implanted with WM983B for MR experiments, 1% isoflurane in 1 L/min oxygen was used to anesthetize the mice. To monitor heart rate, respiration, and core temperature, sub-dermal needle electrodes, a respiration pillow, and a rectal thermistor were used, respectively. Mouse’s core temperature was maintained at 37 ± 1 °C by blowing warmed air into the bore of the magnet with heating controlled by a thermal regulator system. After acquiring the baseline MR spectrum, we injected LND (100 mg/kg) or 0.2 mL of a 300 mg/mL solution of 3-APP (in water; for measurement of pHe)^12^ through two 26-gauge i.p. catheters inserted into either side of the peritoneum without removing the animal from the magnet.

To prepare the LND for injection, a tris/glycine buffer consisting of trizma base (1.2 g) and glycine (5.76 g) in sterile water (100 mL; final pH = 8.3) was prepared^37^. We then dissolved approx. 5 mg LND in tris/glycine buffer (to 22.0 mg/mL), and we vortexed the solution at 40 °C until it was clear.

All MR studies were performed on tumors ~250 mm^3^ in volume and hemispherical in shape. These tumors had sufficient size for optimal spectra and fit the available coils. With this tumor size, we could also obtain a spatially localized spectrum to avoid contamination of tumor metabolites with exogenous metabolites from muscle. pHi, pHe, lactate, and bioenergetics (β-NTP/Pi) data were analyzed using methods as described previously^12,14,15^. pHi and pHe were determined from the Henderson -Hasselbach (pH = pKa + log[α/(1-α)]) where pKa is the dissociation constant of the indicator (Pi or 3-APP, respectively) and α is the degree of ionization of Pi or 3-APP, which can be determined from the chemical shift of these pH indicators. 3-APP is a membrane impermeable phosphate, which is sensitive to the extracellular pH, whereas Pi is assumed to be completely intracellular. The chemical shifts of Pi and 3-APP, were referenced relative to the α-NTP resonance, which is pH insensitive, and the degree of ionization of Pi and 3-APP were measured relative to the limiting chemical shifts determined for complete dissociation of Pi or 3-APP), respectively. For pHe, a pKa value of 6.90 ± 0.03, limiting acid chemical shift of 34.22 ± 0.04 ppm and base chemical shift of 31.08 ± 0.04 ppm were used, and, for pHi, the corresponding parameters were 6.57 ± 0.03, 13.52 ± 0.03 ppm and 11.24 ± 0.02 ppm, respectively^30,38–41^.

The ratio of the peak areas of β-NTP to Pi resonances represents the ratio of high energy phosphates to low energy phosphates, served as an index of the overall bioenergetic status of the tumor. The β-NTP signal is preferably used to determine the β-NTP/Pi ratio since it is not contaminated with signals from ADP, NAD^+^ or NADH, as is the case for α- and γ-NTP peaks^38^.

### Treatment with Doxorubicin and Growth Delay Measurements

WM983B xenografts were grown in nude mice until the tumors reached ~50 mm^3^. At this point, four cohorts of age- and weight-matched mice were assigned to the following treatment groups: Control, LND, DOX, and LND + DOX. The Control group (5 mice) received sham treatment with tris/glycine buffer i.p. and then, 40 minutes later, sham treatment with PBS i.v. The LND group (5 mice) received 100 mg/kg LND i.p. and then, 40 minutes later, sham PBS i.v. The DOX group (5 mice) received sham tris/glycine buffer i.p. and then, 40 minutes later, 10 mg/kg DOX i.v. The LND + DOX group (10 mice) received 100 mg/kg LND i.p. and then, 40 minutes later, 10 mg/kg DOX i.v. We injected the DOX 40 minutes after the LND injection so that the DOX treatment coincided with the tumor’s maximum LND-induced acidification as determined by our MR experiments.

Before all treatment procedures, all mice were anesthetized with 10 mg/mL ketamine hydrochloride and 1 mg/mL acepromazine, and to maintain sedation we gave additional anesthesia every 45–60 minutes. Heating pads were used to maintain body temperature of mice during anesthesia. For the i.v. injections, we restrained the mice using a Mouse Tail Illuminator and then applied tail vein catheters filled with 100 USP Units/mL heparin to prevent blood clotting. For the first five days post-treatment, tumor dimensions and body weight were measured daily with calipers and scale, respectively. After these first five days, these measurements were made every other day. Tumor volume was calculated from its dimensions as described above. Animal studies were performed in accordance with our protocol under the approval of the University of Pennsylvania Institutional Animal Care and Use Committee (IACUC).

Our endpoint for growth delay experiments was four doublings in tumor size (relative to pretreatment volume). Hence, we performed our experiment on the smallest measurable tumor size (50 mm^3^) since we did not want our tumors to exceed 1000 mm^3^ in size.

### Statistics

Data are described graphically and using summary statistics (means, standard deviation (s.d.) or standard error of the mean (s.e.m.). For the experiments to determine whether the minimum value of extracellular acidification differed by cell line and concentration, we used two-way analysis of variance (ANOVA). The two factors were LND dose and time. To adjust for multiple comparisons, Tukey Honest significant difference intervals were used to determine how ECAR_min_ differed as a function of LND concentration.

For the analysis of the NMR measures where the outcome variable was either lactate, β-NTP/P_i,_ pHi or pHe_,_ we fit a two-way repeated measures ANOVA model to the data to account for the effect of repeated measurements of each replicate culture over time. Factors in the ANOVA were cell line and time of observation.

For the analysis of the Seahorse data, basal rates were compared using data from all LND concentrations as these rates were collected prior to LND administration. For the analysis of the ‘stressed’ data, we used only data without LND. Here two-sample *t*-tests were used.

To estimate treatment effects on tumor growth, we conducted a growth delay analysis^42^. For each animal, we recorded the time from treatment until the tumor reached a volume of four times the volume at the initiation of treatment. We then averaged these times in the treated (T) and control arms (C). For the control animals in each line we computed the average slope (B) of the log tumor volume and converted this rate to a doubling time. We estimated the proportion of the tumor cells surviving treatment as e^−(T−C) × B^. We computed 90% confidence intervals by the bootstrap percentile method. We compared the DB-1 and WM983B groups using a two-group *t*-test with standard errors computed using bootstrapping. We note that these methods are slightly different from the original analysis of the DB-1 line^14^. Due to policy changes in our animal facility, the tumor volumes prior to Day 0 were larger for the DB-1 mice than for the WM983B mice. For WM983B, we judged the volume measurements prior to Day 0 to be too noisy to be included in the growth rate analysis. For consistency, we re-analyzed the DB-1 data to ensure that the analyses were consistent for both sets of animals. The original and re-analysis yielded similar, but not identical results. We conducted these analyses in R, Version 3.4.2 R(Foundation for Statistical Computing)^43^.

## Data Availability

All data are available upon request.
